# High-speed multiple-mode mass-sensing resolves dynamic nanoscale mass distributions

**DOI:** 10.1038/ncomms8070

**Published:** 2015-05-12

**Authors:** Selim Olcum, Nathan Cermak, Steven C. Wasserman, Scott R. Manalis

**Affiliations:** 1 Koch Institute for Integrative Cancer Research, Massachusetts Institute of Technology, Cambridge, Massachusetts 02139, USA; 2 Program in Computational and Systems Biology, Massachusetts Institute of Technology, Cambridge, Massachusetts 02139, USA; 3 Department of Biological Engineering, Massachusetts Institute of Technology, Cambridge, Massachusetts 02139, USA; 4 Department of Mechanical Engineering, Massachusetts Institute of Technology, Cambridge, Massachusetts 02139, USA

## Abstract

Simultaneously measuring multiple eigenmode frequencies of nanomechanical resonators can determine the position and mass of surface-adsorbed proteins, and could ultimately reveal the mass tomography of nanoscale analytes. However, existing measurement techniques are slow (<1 Hz bandwidth), limiting throughput and preventing use with resonators generating fast transient signals. Here we develop a general platform for independently and simultaneously oscillating multiple modes of mechanical resonators, enabling frequency measurements that can precisely track fast transient signals within a user-defined bandwidth that exceeds 500 Hz. We use this enhanced bandwidth to resolve signals from multiple nanoparticles flowing simultaneously through a suspended nanochannel resonator and show that four resonant modes are sufficient for determining their individual position and mass with an accuracy near 150 nm and 40 attograms throughout their 150-ms transit. We envision that our method can be readily extended to other systems to increase bandwidth, number of modes, or number of resonators.

## Abstract (web summary)

Nanomechanical resonators are sensitive to tiny changes in their mass. Here, the authors demonstrate a method for quickly measuring many resonator modes and use it to analyse the mass and position of multiple nanoparticles flowing in a fluid channel with a precision of 40 attograms and 150 nm, respectively.

## Graphical abstract

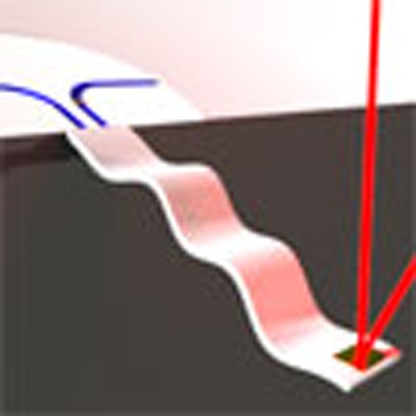

High-quality factors^1^, miniature sizes and small force constants of micro- and nanomechanical resonators have enabled extremely sensitive detection of weak forces^2^, single-molecule interactions^3^,^4^, single-electron spin^5^,^6^, temperature^7^ or mass perturbations^8^,^9^. Most mass sensors detect changes in resonant frequency, a method that has facilitated many exquisite measurements including the weight of single molecules^9^, proteins^10^, exosomes^11^, nanoparticles^11^,^12^, cells^13^,^14^,^15^,^16^ and various accreted chemical analytes^17^. Although these measurements typically exploit perturbations in the fundamental mode frequency, the combined information from multiple modes can yield improved stability^18^ or additional characteristics of analytes. For example, Dohn *et al*.^19^ used multimode measurements to determine the mass and position of attached beads on a microcantilever. Similarly, Hanay *et al*.^10^ measured the mass and position of individual proteins adsorbed on a nanomechanical resonator by measuring the frequency of its first two modes. Beyond mass and position of point masses, multimode measurements have recently been proposed for characterizing continuous mass distributions with atomic-scale resolution^20^, which would be a powerful approach for characterizing biological and synthetic micro- and nanostructures.

However, current systems for multimode frequency measurement are slow, with measurement bandwidths below 1 Hz. Although the speed of open-loop frequency measurements (either thermally or externally driven) are limited by the resonator amplitude timescale^21^, most multimode measurements to date have been performed this way^19^,^20^,^22^,^23^,^24^,^25^,^26^. Existing closed-loop systems also have limited bandwidths (below 1 Hz)^10^. Narrow measurement bandwidths limit throughput—for example, nanomechanical mass spectrometers must measure faster than the time interval between arrivals of individual particles. In addition, wide bandwidths are necessary for resonator sensors that generate fast frequency modulated signals.

Here we introduce a method for wide-bandwidth multimode frequency measurements while oscillating each resonance mode in closed-loop and apply it to measure rapidly changing nanoscale mass distributions. In contrast to previous research exploiting static particles adhered to the surface of a resonator in vacuum^10^,^19^,^22^, we focus on multimode measurements of analytes in motion, while they flow through a suspended nanochannel resonator (SNR)—a vacuum-packaged microcantilever with an embedded fluidic channel^12^ that can measure the masses of nanoparticles^11^. Here, we utilize a scalable system to simultaneously oscillate and track multiple modes of a 200-μm-long SNR in a wide bandwidth. As a demonstration, we track the first four modes to resolve the position and mass of nanoparticle pairs in close proximity as they quickly flow through the resonator. Resolving such closely spaced moving point masses is an important milestone for measuring mass distributions of analytes in solution with high throughput and high resolution.

## Results

### Oscillation scheme

**Figure 1:**
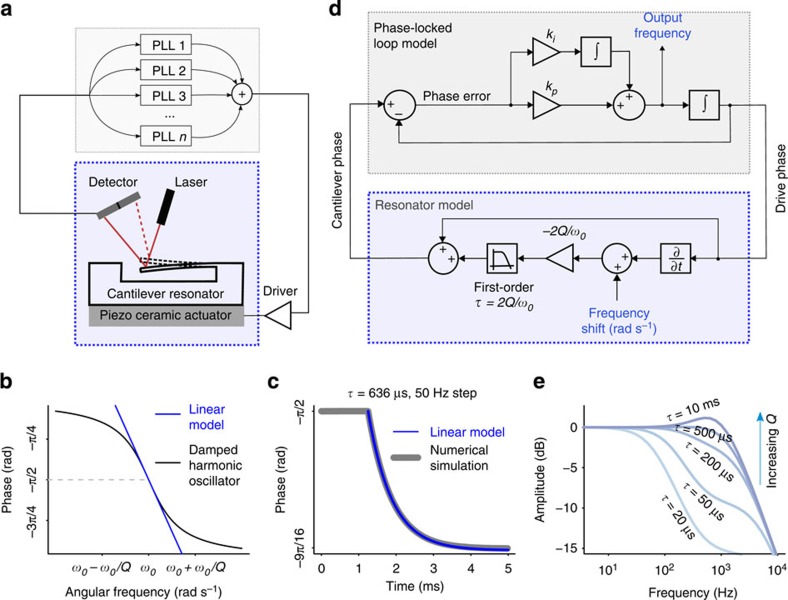
Open loop time constant, *τ* of a harmonic oscillator affects the closed-loop system dynamics. (**a**) Schematic representation of the multimode resonator system operating in closed loop with multiple PLLs (one per resonant mode). (**b**) To make a linear model for the resonator phase response around its resonant frequency, we use the first-order Taylor series of the tangent function and find a slope of −2*Q*/*ω*_0_ (blue line). (**c**) Damped harmonic oscillator phase response to a step in the drive frequency, calculated by time-domain numerical integration (grey line), and a linearized model as a first-order lowpass filter (blue line; see Supplementary Note 1). (**d**) Linear phase-domain model of a resonator-PLL system. The resonator model shown in the blue-dotted box (bottom) is identical to a lowpass filter with a bandwidth of 1/*τ* and a DC gain of zero dB, but depicted differently so that it can provide access to resonant frequency changes as an input (see Supplementary Note 2). Note that the resonator model has a positive feed-forward path that cancels with the PLL's negative feedback path and the PLL integrator cancels with the differentiator in the resonator model (Supplementary Fig. 2a). (**e**) Calculated frequency responses of resonator-PLL systems with varying resonator time constants, *τ*, using the Laplace-domain phase model in **d**. It is evident that at higher frequencies, the resonator-PLL system response is substantially influenced by the resonator quality factor. The results are plotted for a single arbitrarily chosen *k*_p_ and *k*_i_ setting.

In comparison with open-loop techniques, closed-loop approaches in which the resonator is placed in a feedback loop provide wider measurement bandwidths^21^. Furthermore, higher oscillation amplitudes (below the onset of mechanical nonlinearity) lead to reduced frequency measurement noise^27^. For oscillating a single mode, the feedback path typically consists of a phase shift and gain, such that the resonator position signal is delayed, amplified and then fed back to excite the resonator^11^,^28^. This is straightforward to implement and ensures that the loop oscillation frequency nearly instantly follows the resonant frequency^21^. However, for multiple modes it does not allow the phase shift and vibration amplitude for each mode to be separately optimized—a critical requirement for minimizing frequency noise. In contrast, a dedicated phase-locked loop (PLL) in closed loop with each mode as depicted in Fig. 1a allows for setting the phase shift and oscillation amplitude independently.

While separate PLL feedback paths enable independent control over each resonance, they also affect the system dynamics. The ideal resonator-PLL system should track the corresponding resonant frequency as closely and quickly as possible. While direct feedback loops respond to perturbations much faster than the resonator's characteristic amplitude timescale (typically *τ*=2*Q*/*ω*_0_, where *Q* and *ω*_0_ are the quality factor and the angular resonant frequency of the resonator) the case of PLL-mediated feedback^29^,^30^,^31^,^32^,^33^ and its dynamics^34^,^35^,^36^ have been less studied. Therefore, we first developed a Laplace domain model for the resonator-PLL system to understand and then tailor the closed-loop system dynamics.

### Controlling resonator—PLL system dynamics

To model the resonator-PLL system, we first required the transfer function of the resonator's phase. We utilized the time-domain response of a driven harmonic oscillator excited on resonance until time zero and slightly off-resonance after time zero (see Supplementary Note 1). The step change in excitation frequency is conceptually equivalent to instantaneously changing the resonant frequency (for example, by mass adsorption). Approximating the resonator phase delay to be linear around its resonant frequency (Fig. 1b), the first-order Taylor series approximation of the phase term from the time-domain solution reveals that the resonator phase can be well approximated as a first-order low-pass filter in the Laplace domain (Fig. 1c and Supplementary Fig. 1), with a bandwidth equal to 1/*τ*. In the Laplace domain, the transfer function of the resonator phase is:


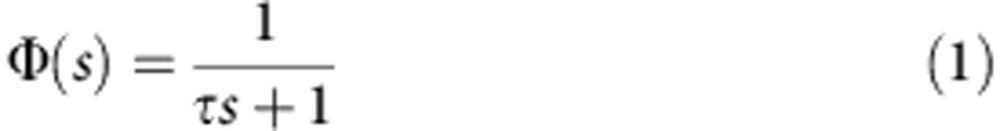


This is valid for frequency steps that are much smaller than 1/*τ*. Figure 1d shows the complete model of a generic resonator-PLL system and suggests that the quality factor of the resonator will substantially influence the loop dynamics especially at high modulation frequencies, demonstrated in Fig. 1e.

For high-precision frequency tracking at high speed, we want each mode's closed-loop transfer function to be maximally flat up to a desired bandwidth. Equating the resonator-PLL transfer function to a Butterworth low-pass filter of desired order and bandwidth yields direct expressions for the PLL parameters to achieve the desired response (Supplementary Note 3). Increasing the PLL order by introducing additional poles in the loop filter (Supplementary Fig. 2b) and using the corresponding parameters in Supplementary Table 1 sharpens the pass-band to stop-band transition (Supplementary Fig. 3). By exploiting this useful relationship, optimally configured resonator-PLL systems can be designed to minimize crosstalk between closely spaced resonant frequencies, such as those that occur in resonator arrays.

### Resonator—PLL system implementation

For realization of a multiple-mode frequency-tracking system, we implemented a scalable array of 12 PLLs in a field-programmable gate array (FPGA) chip, using an architecture similar to other designs (see Methods)^37^. Since the mode frequencies of the SNR are not closely spaced, here we used second order, type 2 PLLs^38^, which can be simplified to first-order low-pass filters when in closed loop with the resonator (first row of Supplementary Table 1 or ref. 34). The implementation of each PLL includes a phase-error detector, a loop filter and a numerically controlled oscillator (see Methods and Supplementary Fig. 2c). Software-programmable parameters in the loop filter control the loop dynamics. To test our system implementation, we measured transfer functions of a PLL alone and a resonator-PLL system over a range of PLL parameters. Across all parameters tested, these transfer functions show excellent agreement with our model predictions for both PLL-only and resonator-PLL cases (see Supplementary Fig. 4 and Supplementary Note 4).

**Figure 2:**
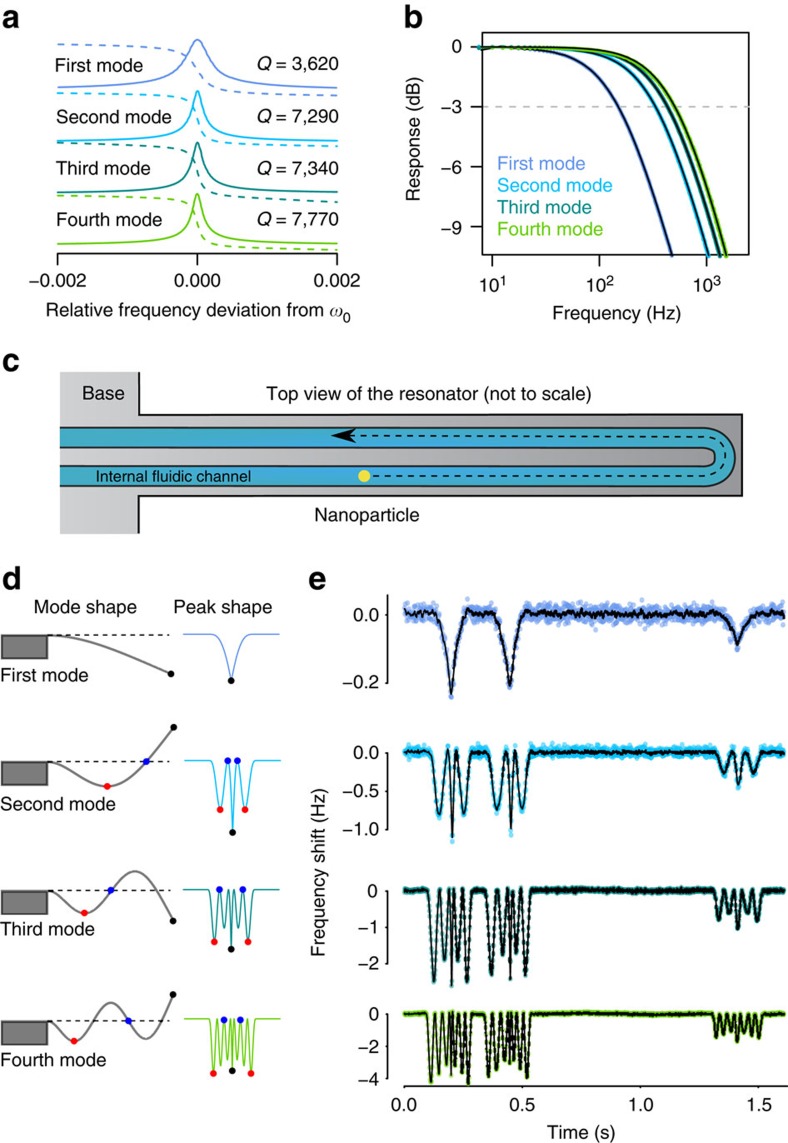
Weighing nanoparticles using multiple vibrational modes of an SNR. After measuring resonant frequencies and quality factors of the first four modes, we set the PLL gains to obtain a flat frequency response with bandwidths between 150 and 500 Hz, and then measure frequency modulation signals generated by nanoparticles passing through the resonator oscillating in four bending modes, simultaneously. (**a**) Transfer function amplitude (solid lines) and phase (dashed lines) for each of the first four bending modes of the resonator measured by a lock-in amplifier. (**b**) Measured frequency responses of the resonator-PLL systems for the four resonant modes (coloured dots) with overlaid predicted first-order transfer functions (black lines). See Supplementary Note 4 for the transfer function measurement method. (**c**) Schematic diagram showing the layout of the internal channel of the resonator along with an example nanoparticle flow path. (**d**) Schematic diagram of the calculated bending profiles of the first four resonant modes (left) along with the corresponding frequency deviations (right) when a particle with constant velocity travels through the resonator similar to that shown in **c**. The locations of several nodes and antinodes are depicted both on the mode profiles and on the frequency modulation signals with matching colours. (**e**) Simultaneous frequency measurements of the first four bending modes as two 150-nm and one 100-nm gold nanoparticle transit through the resonator. Raw data are shown as dots. Because of the lower signal-to-noise ratio in the first and second modes, the overlaid black lines show the data filtered with a 5-point and a 3-point moving average filter, respectively. Black lines for third and fourth modes are unfiltered.

We then placed our PLL array in feedback with an SNR that is 200 μm long, 16 μm wide and 1.3 μm thick with an integrated channel that is 2 μm wide and 700 nm tall. The PLLs excite the resonator modes by driving a piezoceramic actuator seated underneath the chip and an optical lever detector measures the resonator deflection at the tip^11^, which is fed back to the PLLs. The frequencies of the first four modes were 40.48, 249.1, 693.1 and 1,351 kHz, and their quality factors were between 3,500 and 8,000 (Fig. 2a). Beyond the fourth mode, our piezoceramic was not able to actuate the resonator with sufficient amplitude. We configured the closed-loop frequency response of each mode to behave as a first-order low-pass filter (Fig. 2b) by setting the loop parameters using Supplementary Table 1. The bandwidth for each mode was chosen to be wide enough for resolving particles with >100-ms-transit time and ranged from 150 to 500 Hz (see Supplementary Fig. 5 and Supplementary Note 5).

### Nanoparticle mass distribution measurements

**Figure 3:**
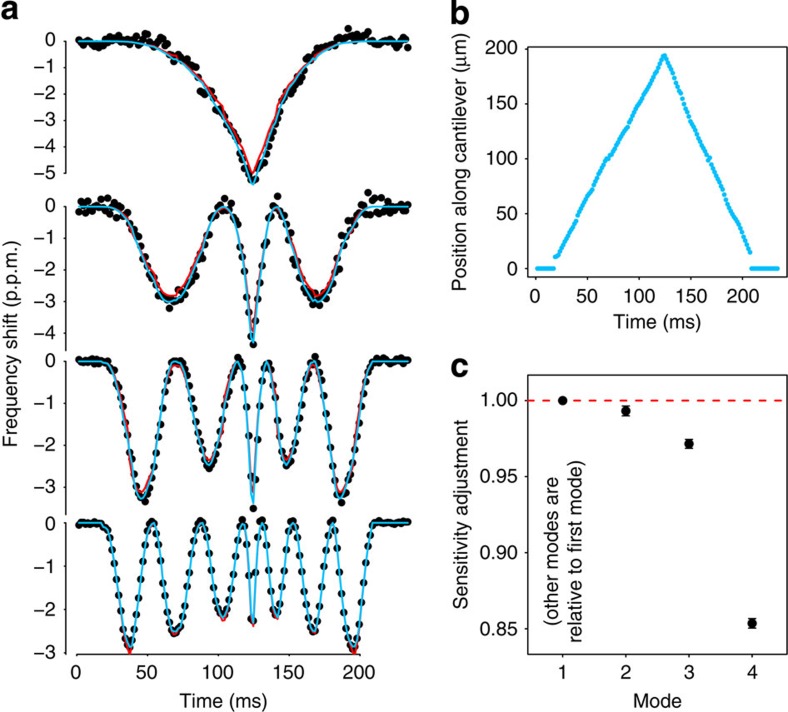
Multimode measurements yield the trajectory of a flowing nanoparticle. (**a**) Four mode data for a single particle (black points) fit to the expected peak shape^22^ when the particle has a fixed mass and can be at any position at any time (red lines), and when the mode sensitivities are allowed to vary as well (blue lines). Note the systematic deviations of the red fits at the local minima for each signal. (**b**) Position of the particle over time, estimated from the data in **a**. (**c**) Estimated sensitivities of other modes compared with the expected values. Points and error bars are the mean and s.e.m. of 31 150-nm gold particles, each of which were fit individually to a model, in which the mode sensitivities were allowed to vary relative to the expected values.

Next, we measured the resonant frequency signals of all four modes while a mixture of 100 and 150 nm gold nanoparticles flowed through the resonator (Fig. 2e). As particles typically took longer than 100 ms to transit the resonator, we averaged and downsampled the signals to a sampling rate of 642 Hz, yielding acquisition bandwidths between 150 and 285 Hz. We fit the resulting frequency signals to a model of a point loading on a cantilever^22^ to obtain single particle mass and trajectory information (see Methods). The model assumed that the particle could be in any position at any time (that is, one free parameter for each time point) and that particle mass is constant during transit. The data systematically deviated from the model in that the magnitudes of frequency shift of modes two, three and four were smaller than predicted (Fig. 3a). Empirically, adding sensitivity-adjustment parameters for modes two through four significantly improved the fit (Fig. 3a, blue curves) and reduced the root-mean-square (RMS) error by 25% with only three additional degrees of freedom. An example of a best-fit particle trajectory is shown in Fig. 3b. The mean estimated sensitivity adjustment factors obtained by fitting 31 150-nm particles are shown in Fig. 3c. We are uncertain as to the origin of these deviations, although they are not attributable to undesired smoothing via insufficient bandwidth.

**Figure 4:**
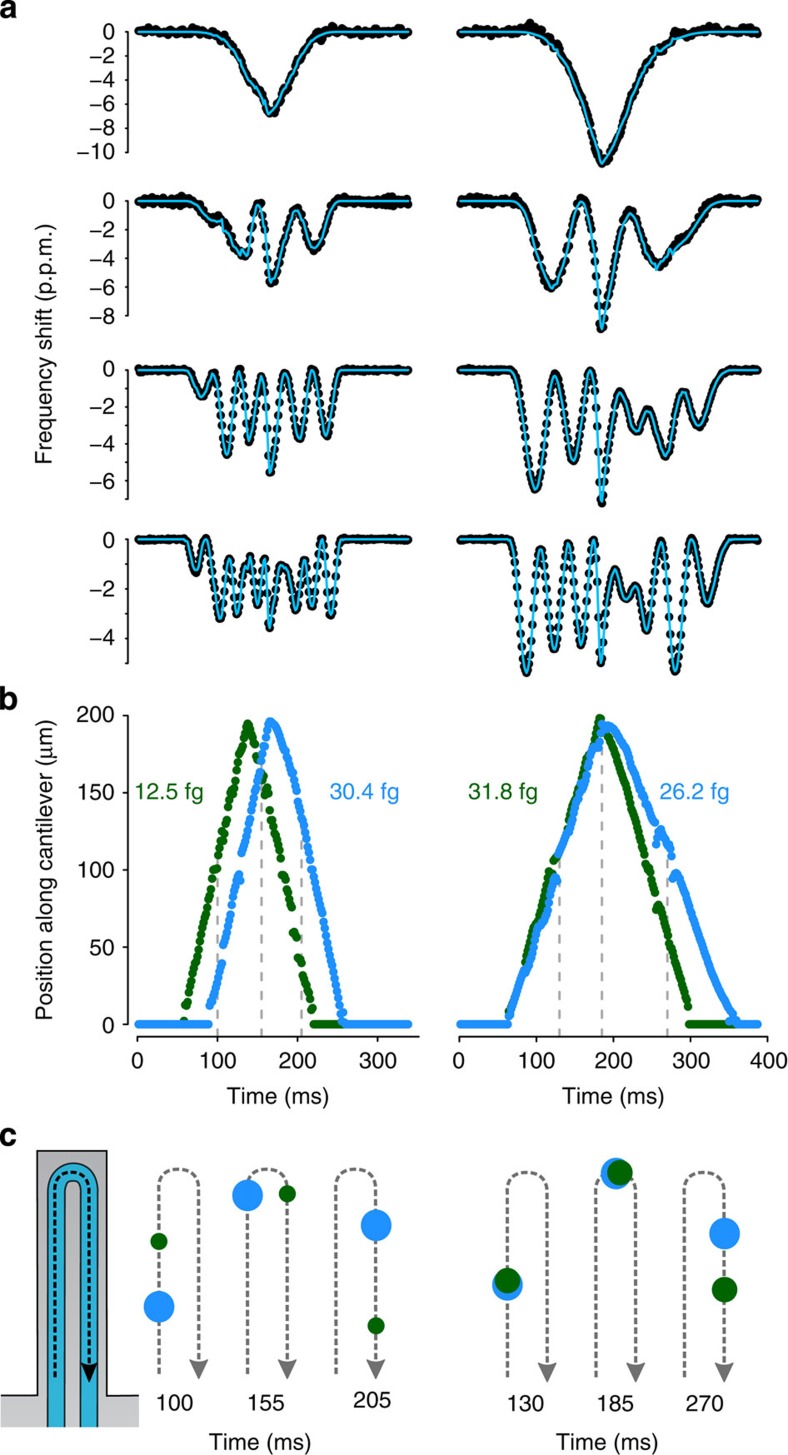
Fast multimode measurements resolve dynamic distribution of nanoparticle masses. (**a**) Four mode data (black dots) from two instances in which two particles nearly simultaneously traversed the resonator, and a fit to a model in which two particles are simultaneously present (blue lines). (**b**) Calculated masses and positions of each particle during their transits in the resonator as a function of time. (**c**) Illustration showing particle locations at varying time points (noted as dashed lines in **b**) as they flow through the resonator (not to scale).

As a first step towards obtaining mass distributions within a microfluidic channel, we demonstrate the capability of our system to simultaneously extract mass and position of nanoparticle pairs flowing through the resonator. As shown in Fig. 4a, a single mode provides limited information about the particles. However, by utilizing all four modes it was possible to resolve the position and mass of both particles as they flowed through the resonator (Fig. 4b). The first example shows two particles following each other in the channel (illustrated in Fig. 4c). We can see that at the tip of the resonator the heavier particle slows down because of higher centrifugal force opposite to the direction of the flow^12^. The second case shows two particles following each other very closely in the first half of the channel. When the particles turn at the tip of the resonator, one of them veers away from the initial flow path to a path where the flow velocity is slower.

### Noise analysis

**Figure 5:**
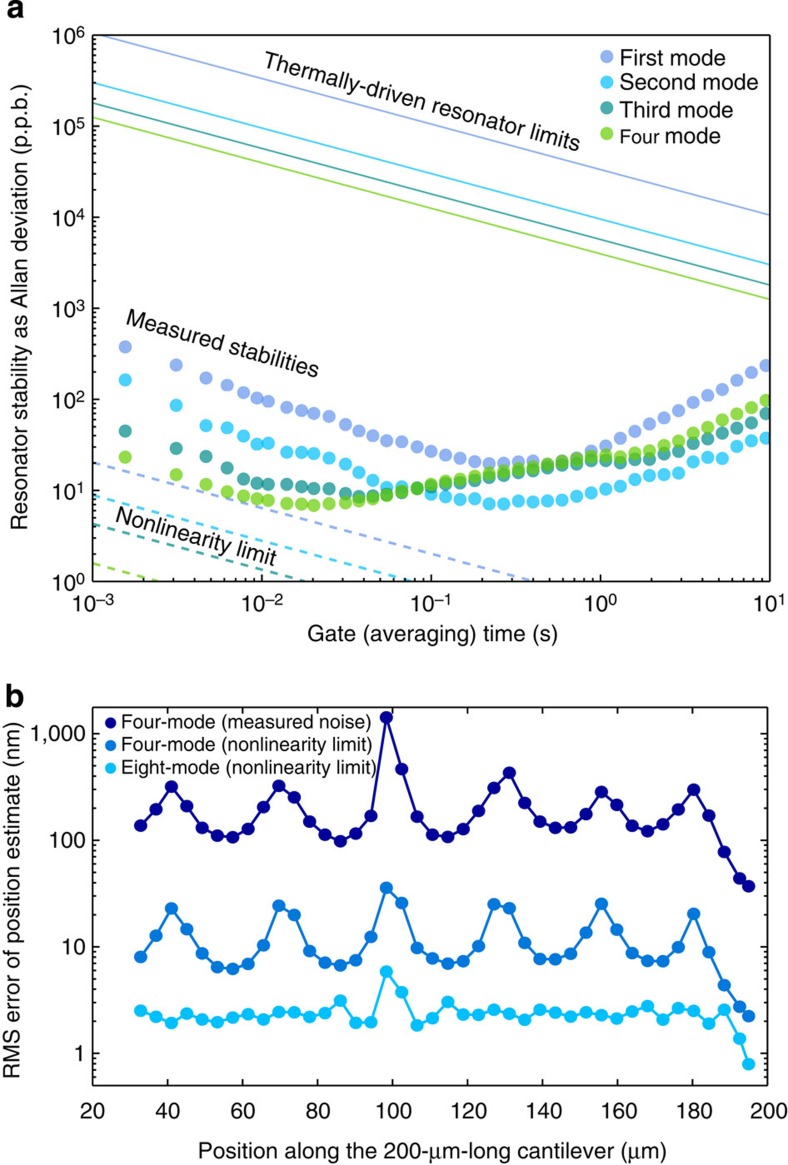
Multimode frequency stability limits mass position precision. (**a**) Measured fractional Allan deviations of four modes of the resonator (coloured circles), while they are simultaneously oscillated, as a function of averaging times. For comparison, we also show the theoretical noise limits for this cantilever when thermally driven (solid lines) or individually driven to the onset of nonlinear behaviour (dashed lines). (**b**) Root-mean-square error of the position estimation of a 30-fg (∼150-nm gold) particle as a function of its position along the resonator length. The error is calculated by running the fitting algorithm on simulated signals of identical particles passing through the resonator in 150 ms corrupted with experimentally measured noise (dark blue), thermal noise when maximally driven in the first four modes (blue) and thermal noise when maximally driven in the first eight modes (teal).

The precision of our position and mass estimates will depend on the noise properties of the modal measurements. To assess this, we simultaneously recorded 1-min noise waveforms from each mode and found that all the modes exhibited minimum Allan deviations at gate times between 20 and 500 ms, ideal for fast particle measurements (Fig. 5a, coloured circles). The measured minimum fractional Allan deviations range from 7 to 19 p.p.b., which are more than 3,000-fold lower than what would have been achievable for thermally driven (free-running) resonators (Fig. 5a, solid lines). However, if we could improve the dynamic range of our detector such that all modes could be oscillated at the onset of mechanical nonlinearity (measured here as 94, 91, 92 and 97 dB above the thermal fluctuations), we could improve the frequency stabilities by over an order of magnitude (Fig. 5a, dashed lines). Calculation details for Allan deviation and thermal noise limits are provided in Methods and in ref. 18.

As the nanoparticles are inside an opaque silicon beam, we cannot visually observe their location for comparison against our measurements. However, assuming a well-validated model of how point-mass loading affects modal frequencies^19^,^22^, we can estimate the precision using the measured frequency noise. To determine the precision of the position estimation due to frequency noise alone, we fit a set of model-generated frequency signals corrupted with the recorded waveforms of frequency noise. We first generated the four-mode frequency modulation signals for a 150-nm gold nanoparticle (30 fg) making a 150-ms transit through the resonator. We then randomly sampled 250 short contiguous subsets from our noise measurements, added each to the theoretical particle waveforms and solved for the mass and positions to obtain their standard errors. This explicitly takes into account the frequency spectrum of our noise. In our case of four modes, we can determine the position of a 150-nm particle with a typical RMS error of 152 nm along the length of the resonator and 37 nm at the tip (Fig. 5b, black circles). While this position precision will improve with increasing particle mass, the uncertainty of the mass estimate (41 attograms) is independent of particle mass. In addition, while we simulate a 150-ms particle transit, we estimate a position for each time point, rendering the position precision effectively independent of the transit time.

Although previous work has questioned the value of extra higher-order modes^22^, we compared our position precision using four modes against using only the first two or three modes and found a notable improvement with each extra mode introduced (Supplementary Fig. 6a). Similarly, achieving thermally limited noise while driving eight modes just below the onset of nonlinearity would improve the position precision by two orders of magnitude (Fig. 5b). Even greater gains could be achieved by using smaller resonators—a 10-fold shorter SNR similar to those in ref. 11 with similar stabilities in eight modes would enable analysing single virions or extracellular vesicles (∼100 nm) with 4-nm position precision (see Supplementary Note 6 and Supplementary Fig. 6b). However, smaller resonators come at the cost of high resonant frequencies, requiring specialized actuation and detection schemes. To operate smaller resonators, future systems may employ optimized piezoresistor sensors^39^,^40^,^41^ and alternate optical^42^,^43^,^44^ or electrical^45^,^46^,^47^ low noise transduction schemes that can sustain multiple resonances at high frequencies^23^.

## Discussion

The ability to resolve nanoparticle pairs in close proximity in a resonator suggests the possibility of observing bimodal mass distributions within a population of single particles, or resolving high-aspect-ratio shapes versus more spherical shapes in solution. In addition, to be able to monitor the dynamic changes in the mass distributions could be used for online monitoring of the assembly of engineered nanoparticles such as DNA origami or nanoparticles designed for nanomedicine. The ability of mechanical resonators to study the distribution of mass in analytes will ultimately depend on the resonator size, the number of modes measured and the frequency measurement precision. Improvements in any/all of these three areas, perhaps in parallel with mass tagging at specific locations, could ultimately enable analysing the structure of lighter biological particles such as phage, viruses or exosomes in solution, or single proteins with nanomechanical mass spectroscopy in vacuum, which are currently difficult to resolve with optical techniques and laborious to observe with other methods. The same approach, when applied to larger resonators that can sustain bacteria or mammalian cells, could ultimately be used to obtain high-throughput mass tomography of single living cells.

We believe that the presented method will also prove useful for large-scale integration of resonators. A carefully configured PLL array can oscillate an array of resonators individually with combined-detector and combined-actuator signals. The presented ability of engineering the system responses of individually addressable (by resonant frequency) resonators will be pivotal to such large-scale implementations. Such an approach could enable systems exploiting resonators ranging from very sensitive gas sensors to artificial nose applications to high-throughput cell analysers.

## Methods

### Device fabrication

The SNR used in the experiments was fabricated using a microfabrication process^12^,^13^ that was carried out at Innovative Micro Technologies, Santa Barbara, CA. The device includes a hollow microcantilever free to oscillate in a vacuum-sealed cavity with an on-chip getter, enabling long-term high-quality factor operation. The embedded fluidic channel in the SNR is accessed from the two sides by two larger (50 μm by 20 μm) bypass channels, which have two fluidic ports each. The top-side of the device is protected by a glass wafer, which enables optical access to the resonator.

### PLL implementation

The PLL was implemented on an Altera Cyclone IV FPGA on a DE2–115 development board from Terasic Technologies. The board clock signal was generated by a 100-MHz oven-controlled crystal oscillator (Abracon AOCJY2). Analogue-to-digital and digital-to-analogue data conversion were performed using a daughter board from Terasic with two A/D converters and two D/A converters, all 14-bit and running at 100 MHz, connected to the FPGA via a high-speed mezzanine connector. The FPGA code was written in Verilog and compiled using Quartus II 12.1 (Altera). The code includes 12 identical PLL modules. Each module utilizes a multiplier and a low-pass filter as a phase error detector (Supplementary Fig. 2c). The low-pass filter is a second-order cascaded integrator-comb-decimating filter^48^, with a variable rate change factor (and thus variable bandwidth). Unfortunately, this phase detector is sensitive to both the phase and the amplitude of the input:


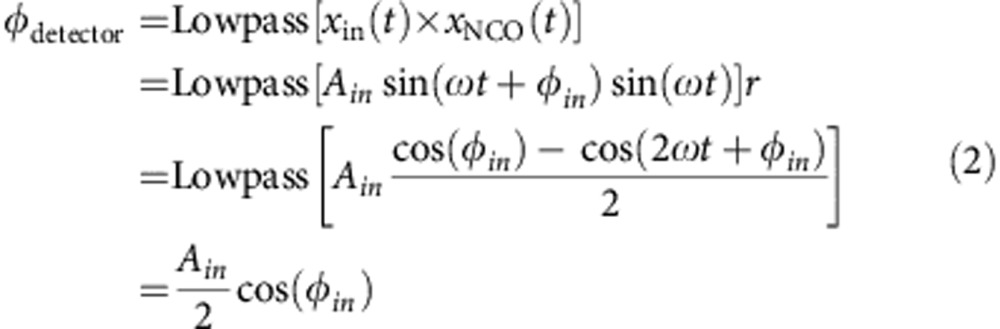


The phase detector is therefore linear around *φ*_in_=−*π*/2, where the PLL locks; however, it has a gain that depends on the input signal amplitude. Therefore, we calculate the input amplitude *A*_in_ and divide the phase error by it. This is implemented by multiplying the input signal by both the sine and cosine outputs of the numerically controlled oscillator (NCO), low-pass-filtering both and calculating the sum of the squared values, yielding the input magnitude squared. We then use a look-up table to calculate an appropriate fractional gain to cancel out the input magnitude, as both square root and division operations are logic-intensive and slow, often not meeting timing requirements. This normalized error signal is then split into two paths, one of which is rescaled and integrated (with some finite frequency bounds outside which the integration saturates, so that the PLL cannot accidentally lock to other modes), and the other of which is rescaled and then summed with the integral path. This signal is then fed into a 35-bit NCO with a frequency resolution of 2.9 mHz.

Each PLL module is connected to a 32-bit CPU implemented on the FPGA (Nios II, Altera). The CPU can both write to control registers inside the PLL to set parameters such as the proportional or integral gains or the output drive amplitude, as well as read various PLL state variables such as the current NCO frequency. In our system, the DE2–115 board is connected to a computer via gigabit ethernet, and C code running on the Nios II processor creates a simple server through which the PC can connect and read/write PLL registers. Writing to registers occurs over a TCP connection to ensure reliability; however, the NCO frequency is transmitted from the FPGA via UDP multicast, allowing for much lower overhead and higher bandwidth. We find that we can easily transmit uncompressed frequency data (32-bit integers) at a rate of over 100 kHz with no dropped packets. On the PC, we have implemented a LabView (National Instruments) software to save this data stream, as well as let us easily set the PLL control register values over the TCP connection.

In the current configuration, each PLL module takes up roughly 6,500 logic cells (out of 114,480), 11 18 × 18-bit multipliers (out of 266 total), and four M9K memory blocks (out of 432 total) of an Altera Cyclone IV FPGA on a DE2–115 development board. As the CPU takes up roughly 15,000 logic cells, two 18 × 18-bit multipliers, and 253 M9K memory blocks, logic cells are the limiting factor in increasing the number of parallel PLLs running on a single FPGA. In the current implementation, we can fit 14–15 PLLs on our FPGA though future implementations with higher-end FPGAs could fit many more—for example, the Altera Stratix III EP3SL340 could likely fit around 50 PLLs.

### Single-particle fits

We rely on the following equation given in ref. 22 relating the relative frequency shift 
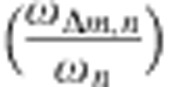
 of a resonator of mass *m*_0_ operating in mode *n*, when loaded with a point mass Δ*m* at a position *z*:


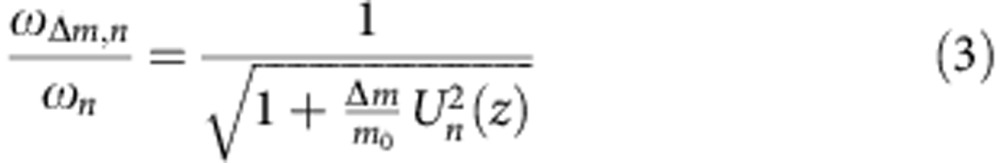


where *U*(*z*) is given by


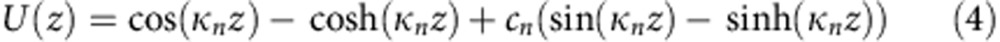


with *κ*_*n*_ being the *n*th root of cos(*x*) cosh(*x*)=−1 and *c*_*n*_=(sin(*κ*_*n*_)−sinh(*κ*_*n*_))/(cos(*κ*_*n*_)+cosh(*κ*_*n*_)). In this case, *z* is parameterized such that 0 represents the base of the cantilever and 1 is the tip of the cantilever.

To account for the reduced sensitivity in higher modes (as compared with what is expected in (2)), we modify this model slightly by including a sensitivity adjustment parameter *s*_*n*_, which is fit for all modes except the first (*s*_1_ is defined as 1).


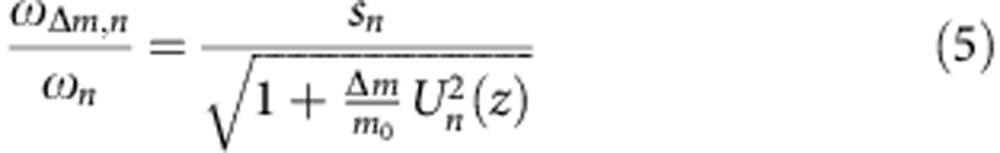


To fit particle trajectories, we follow ref. 22 by attempting to minimize the residual squared error *χ*^2^ of the normalized signals (fit errors are divided by the s.d. of the signal, such that a unit residual error is equally penalized for all modes). Free parameters in this fit are noted in red:


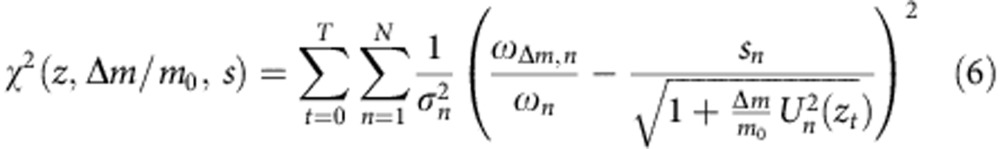


Here *z* is a vector consisting of one value per time point *t*, and is not constrained based on expected flow path. *T* is the number of timepoints, *t* indexes the timepoints, *N* is the number of modes, *n* indexes the modes and *σ*_*n*_ is the RMS noise in mode *n*.

### Two-particle fits

We proceed in a very similar manner for the two-particle fits, minimizing the following objective function^19^:


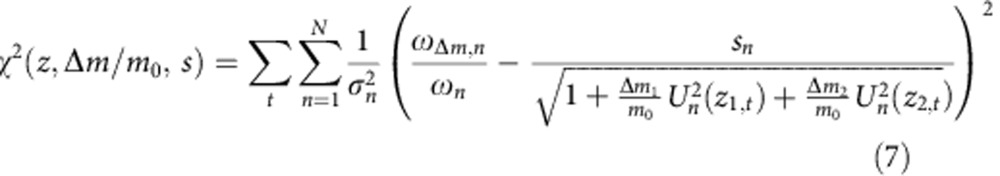


### Noise analysis calculations

The fractional Allan deviation, *σ*_*A*_(*τ*_*A*_), of the frequency of an oscillator in a time period of *τ*_A_ is defined as in ref. 49:


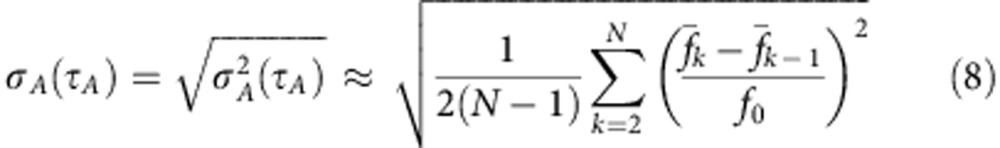


where 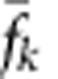 is the time average of the frequency measurement in the *k*^th^ time interval of length *τ*_A_ within a total of *N* intervals, and *f*_0_ is the mean oscillation frequency calculated over the entire duration of the noise measurement. The fractional Allan deviation of a resonator due to thermal noise is given as^18^:


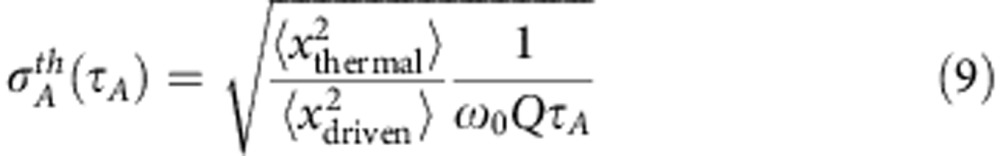


where 
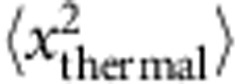
 is the mean squared displacement because of thermal vibration, and 
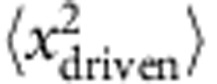
 is the mean squared displacement when driven. For a purely thermally driven cantilever, the ratio of these quantities is one.

## Author contributions

All authors contributed to the design of the study and writing of the manuscript. S.O. and N.C. performed the experiments and analysed the data.

## Additional information

**How to cite this article:** Olcum, S. *et al*. High-speed multiple-mode mass-sensing resolves dynamic nanoscale mass distributions. *Nat. Commun.* 6:7070 doi: 10.1038/ncomms8070 (2015).

## Supplementary Material

Supplementary Information Supplementary Figures 1-6, Supplementary Table 1, Supplementary Notes 1-6, and Supplementary References
