# Supplementary material for: High-speed multiple-mode mass-sensing resolves dynamic nanoscale mass distributions
Source: Nat Commun. 2015 May 12;6:7070. doi: 10.1038/ncomms8070 (PMC4432639; doi:10.1038/ncomms8070)
Supplement: Supplementary Information — Supplementary Figures 1-6, Supplementary Table 1, Supplementary Notes 1-6, and Supplementary References [file ncomms8070-s1.pdf]

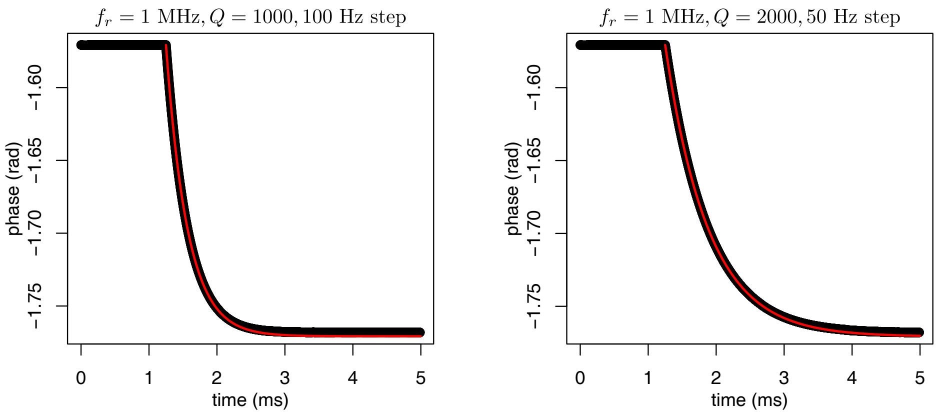

Supplementary Figure 1: Resonator phase response to a step change in the drive frequency. Time-domain simulation (forward Euler) of the phase response of a resonator to a step in the drive frequency (black), overlaid with the linear model prediction from equation (S8) in red.

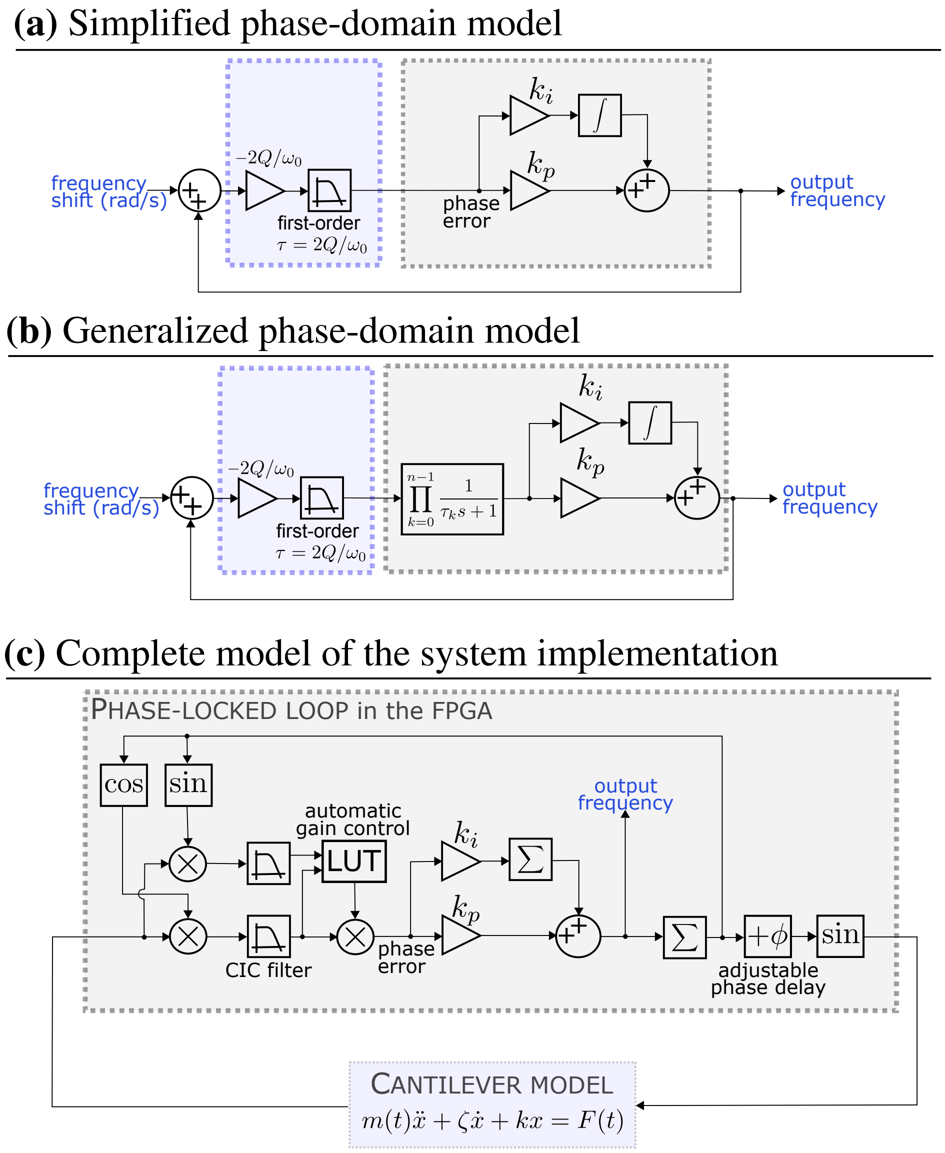

Supplementary Figure 2: Block diagram representations of the models used to analyze a resonator-PLL loop. (a) Phase-domain block diagram of simplified resonator-PLL closed loop system. The integrator representing the VCO in Figure 1d is cancelled by the differentiator of the resonator. **(b)** Phase-domain block diagram of the same system in (a) with $n-1$ poles inserted in the forward path. The additional poles increase the order of the characteristic polynomial of the closed loop transfer function for achieving n^{th} order Butterworth filter response. **(c)** Complete block diagram representation of one element of the digital PLL array implemented on the FPGA.

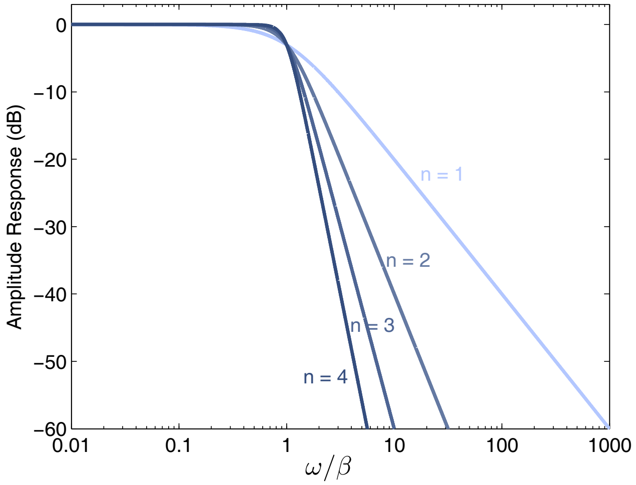

Supplementary Figure 3: PLL parameters can be set to tailor closed loop dynamics. Amplitude response of the closed loop system as a function of frequency normalized to desired bandwidth, β when loop parameters given in Supplementary Table 1 are evaluated in (S13). The calculated responses are identical to that of a Butterworth low-pass filter of given order.

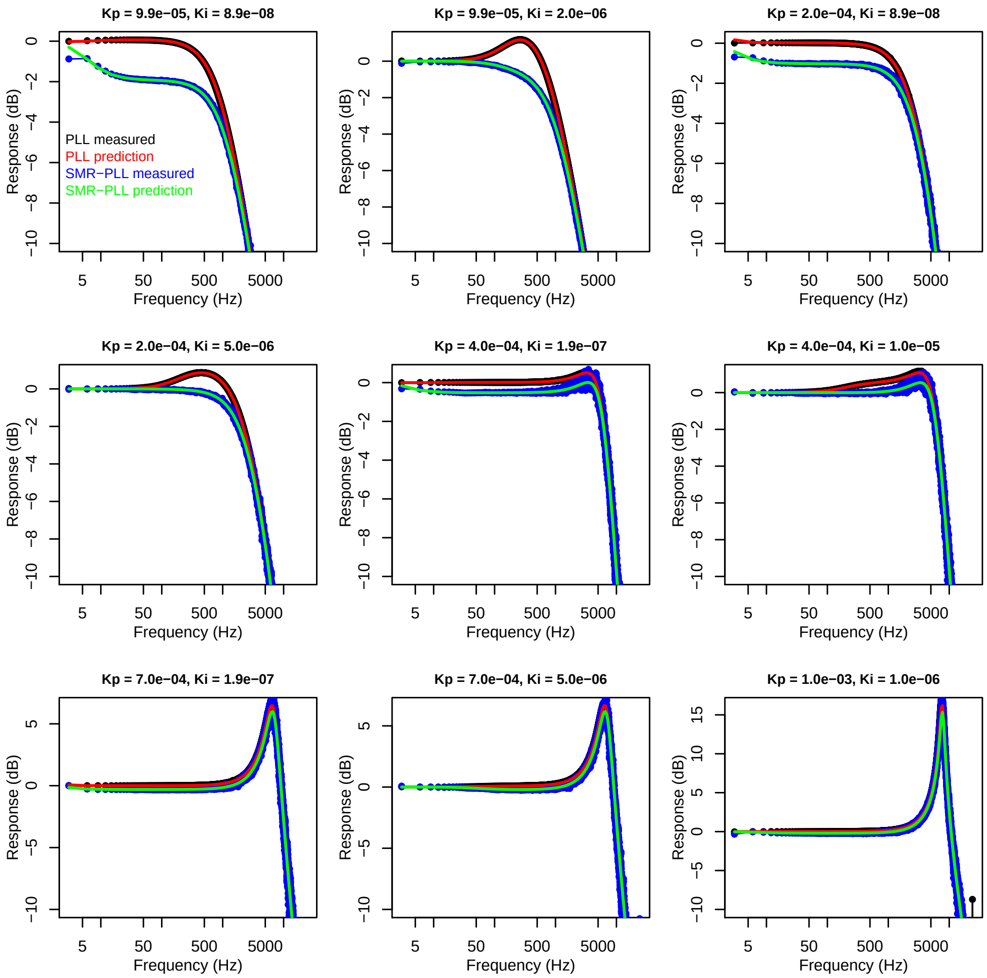

Supplementary Figure 4: Comparison of measured versus modeled SMR and SMR-PLL transfer functions across varying PLL settings. Calculated versus measured transfer functions for PLL alone (red and black) and SMR-PLL system (green and blue). The resonator was a 160 μm-long cantilever with a 3×5 μm embedded channel and a Q factor of approximately 2800. For the above plots, the CIC filter had rate change factor $R = 2048$.

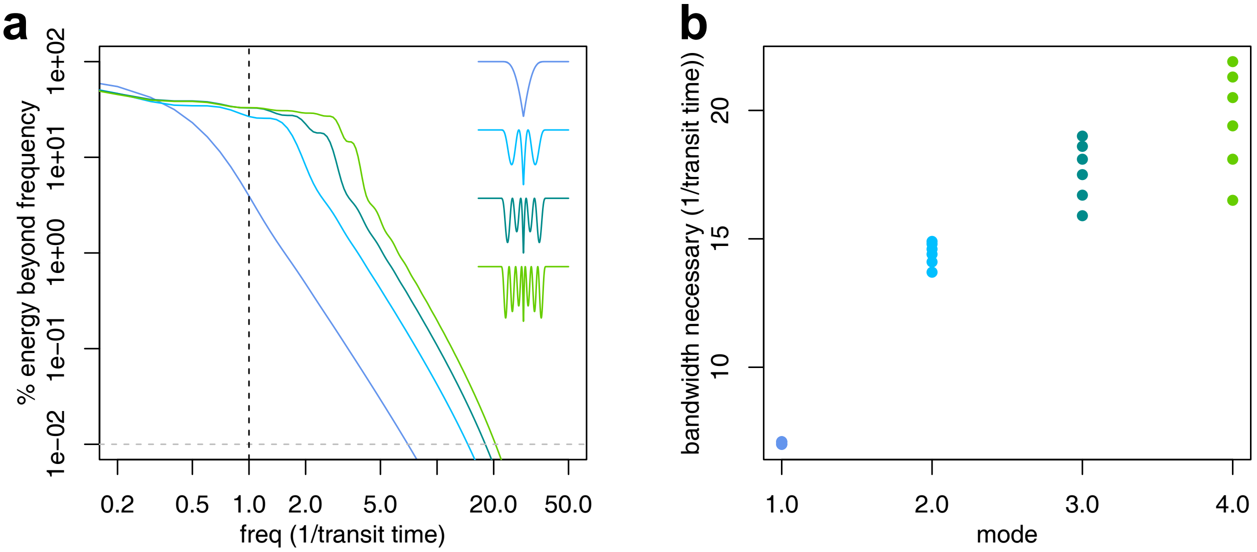

Supplementary Figure 5: Spectral content of a particle's (demodulated) signal by mode. **(a)** One minus integral of the energy spectral density, or what fraction of the signal energy is being lost if we exclude all energy at higher frequencies. Different colors represent increasing modes, for which the peak shapes are shown inset. **(b)** Required measurement bandwidth for recovering >99.99% of the energy, as a function of mode. Signal bandwidth depends on particle path - different dots for a single mode represent particles transiting 95%, 96%, 97%, 98%, 99% or 100% of the length of the cantilever. Cantilever resonant frequency signal shapes are calculated based on reference [1].

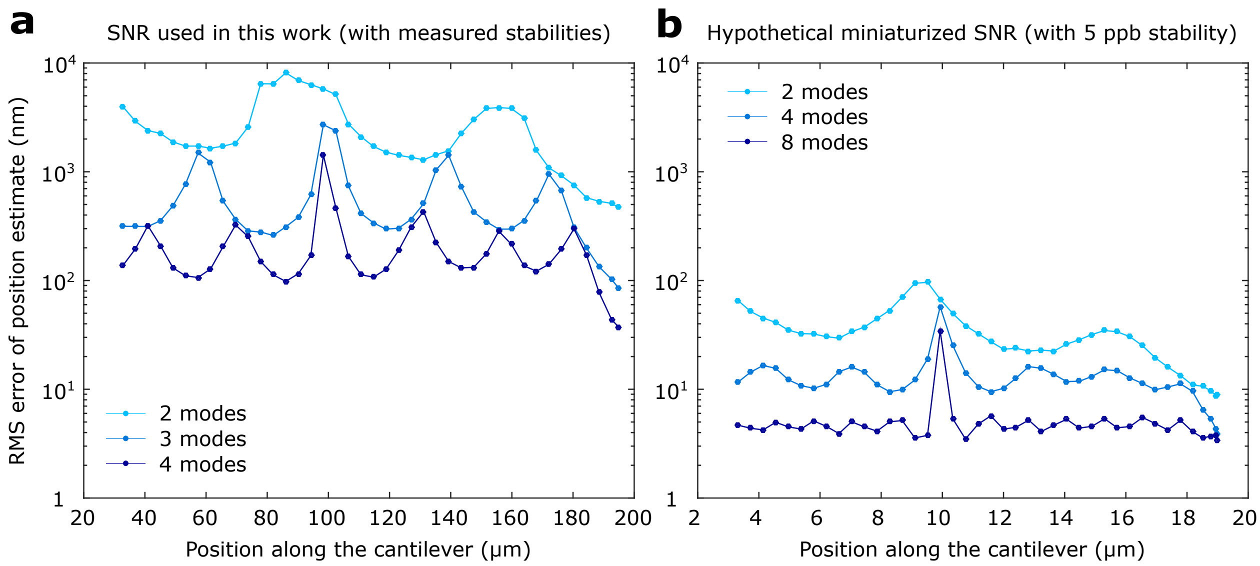

Supplementary Figure 6: Miniaturization and/or additional modes and improve position precision, i.e. RMS error of the position estimation. **(a)** The position precision of the SNR used in this work when the first two, three and four modes are used to weigh a 30-femtogram gold nanoparticle (150 nm). The curve for four modes on this plot is identical to the 4-mode curve in Figure 5. **(b)** The position precision of a hypothetical miniaturized SNR weighing a 80-attogram human immunodeficiency virus ($\sim$100 nm) when first two, four and eight modes are oscillated with a frequency stability of 5 ppb.

Supplementary Table 1: Resonator-PLL Loop parameters for achieving n^{th} order Butterworth filter response.

n	k_p	k_i	τ_1	τ_2	Additional Poles
1	β	k_p/τ			0
2	$\beta/\sqrt{2}$	k_p/τ	$\sqrt{2}/2\beta$		1
3	$\beta/2$	k_p/τ	$(1 \pm j)/2\beta$	$(1 \mp j)/2\beta$	2

Supplementary Note 1 Phase-domain transfer function

As noted in the main text, for deriving the transfer function of the phase of a resonator, we analyze its time-domain response to an abrupt change in the excitation frequency. Prior to the frequency change, we assume that the resonator is oscillating in steady state with amplitude $A(\omega_0)$ and phase-delay $\theta(\omega_0)$ as given in references [2, 3],

$$A(\omega) = \frac{F_0/m}{\sqrt{(\omega_0^2 - \omega^2)^2 + (\omega_0\omega/Q)^2}} \tag{S1}$$

$$\theta(\omega) = \arctan\left(\frac{\omega_0\omega}{Q(\omega^2 - \omega_0^2)}\right) \tag{S2}$$

where F_0 is the amplitude of the driving force, m is the effective mass of the resonator, ω_0 is the resonant frequency and Q is the quality factor of the resonator. When the excitation instantaneously changes its frequency, the resonator response is described by the sum of the zero-input response and the zero-state response as follows:

$$x(t) = A(\omega_0)e^{-t/\tau}\sin(\omega_0 t + \theta(\omega_0)) + A(\omega_1)(1 - e^{-t/\tau})\sin(\omega_1 t + \theta(\omega_1)) \tag{S3}$$

where ω_1 is the new excitation frequency and $\tau = 2Q/w_0$. If we write (S3) in the form of $x(t) = A(t)\sin(\omega_1 t + \phi(t))$, $\phi(t)$ gives the phase of the resonator as a function of time. Therefore, we will use the following identity

$$a\sin(\psi_1) + b\sin(\psi_2) = c\sin\left(\psi_1 + \arctan\left[\frac{b\sin(\psi_2 - \psi_1)}{a + b\cos(\psi_2 - \psi_1)}\right]\right) \tag{S4}$$

We will entirely ignore the amplitude term c, other than noting that it is a time-dependent function. We'll use the following definitions to coerce (S3) into the form of (S4):

$$\begin{aligned}
a &= A(\omega_1)(1 - e^{-t/\tau}) \\
b &= A(\omega_0)e^{-t/\tau} \\
\psi_1 &= \omega_1 t + \theta(\omega_1) \\
\psi_2 &= \omega_0 t + \theta(\omega_0) \\
\alpha &= \psi_2 - \psi_1 = (\omega_0 - \omega_1)t + \theta(\omega_0) - \theta(\omega_1)
\end{aligned} \tag{S5}$$

Therefore we are left with:

$$x(t) = c(t)\sin\left(\omega_1 t + \theta(\omega_1) + \arctan\left[\frac{A(\omega_0)e^{-t/\tau}\sin(\alpha)}{A(\omega_1)(1 - e^{-t/\tau}) + A(\omega_0)e^{-t/\tau}\cos(\alpha)}\right]\right) \tag{S6}$$

Multiplying the *arctan* argument by $e^{t/\tau}/e^{t/\tau}$ and dividing by $A(\omega_0)/A(\omega_0)$, we get:

$$x(t) = c(t)\sin\left(\omega_1 t + \theta(\omega_1) + \arctan\left[\frac{\sin(\alpha)}{\frac{A(\omega_1)}{A(\omega_0)}(e^{t/\tau} - 1) + \cos(\alpha)}\right]\right)$$

Define $\tilde{\alpha} \equiv \theta(\omega_0) - \theta(\omega_1)$, and note that $\frac{A(\omega_1)}{A(\omega_0)} = \frac{\omega_0}{\omega_1}\cos(\tilde{\alpha}) \approx \cos(\tilde{\alpha})$, where the approximation is valid for very small frequency deviations.

$$x(t) = c(t)\sin\left(\omega_1 t + \theta(\omega_1) + \arctan\left[\frac{\sin(\alpha)}{\cos(\tilde{\alpha})e^{t/\tau} - \cos(\tilde{\alpha}) + \cos(\alpha)}\right]\right)$$

If the the frequency step $\omega_0 - \omega_1$ is very small, then $(\omega_0 - \omega_1)t$ is a very slow term compared to $e^{t/\tau}$, and we can approximate the time-varying α as the constant $\tilde{\alpha}$. Then the equation reduces to:

$$x(t) = c(t)\sin\left(\omega_1 t + \theta(\omega_1) + \arctan\left[\tan(\tilde{\alpha})e^{-t/\tau}\right]\right)$$

The Taylor expansion of $\arctan(\tan(\alpha)x) = x\tan(\tilde{\alpha}) - \frac{1}{3}\tan^3(\tilde{\alpha})x^3 + O(x^5)$. Noting that $\tilde{\alpha}$ is typically small justifies dropping all but the linear term, yielding

$$x(t) = c(t)\sin\left(\omega_1 t + \theta(\omega_1) + \tan(\tilde{\alpha})e^{-t/\tau}\right)$$

Approximating $\tan(x)$ with x

$$\begin{aligned} x(t) &= c(t)\sin\left(\omega_1 t + \theta(\omega_1) + (\theta(\omega_0) - \theta(\omega_1))e^{-t/\tau}\right) \\ &= c(t)\sin\left(\omega_1 t + \theta(\omega_0) + (\theta(\omega_1) - \theta(\omega_0))(1 - e^{-t/\tau})\right) \end{aligned} \tag{S7}$$

Noting that $\theta(\omega)$ is also a *tan* function, we approximate that with its 1st-order Taylor series around the resonant frequency, $\theta(\omega) = \theta(\omega_0) - \tau(\omega - \omega_0)$:

$$x(t) = c(t)\sin\left(\omega_1 t + \theta(\omega_0) - \tau(\omega_1 - \omega_0)(1 - e^{-t/\tau})\right) \tag{S8}$$

To verify that our approximations were reasonable, we compared our model predictions to time-domain simulations of a driven damped harmonic oscillator that experiences a step in the drive frequency. In our simulation, we estimate the phase delay based on the timing of zero-crossings (strictly, only zero-crossings where the signal is increasing). Defining the vector of resonator zero-crossing times as t_{r0} and the vector of drive zero-crossing times as t_{d0}, we calculate the phase at every resonator zero-crossing as $\phi = \frac{t_{r0}[n] - t_{d0}[n]}{t_{d0}[n] - t_{d0}[n-1]}$. The results of this simulation, plotted as the black curves in Supplementary Figure 1, agree very well with the approximation in equation (S8) plotted as the red curves.

For calculating the phase domain transfer function of a resonator, we used the approximate expression in (S8), where the response of the resonator phase, $\phi(t)$, to a phase step of $-\tau(\omega_1 - \omega_0)$ at $t = 0$ is given as:

$$\phi(t) = \theta(\omega_0) - \tau(\omega_1 - \omega_0)(1 - e^{-t/\tau}) \tag{S9}$$

Note that $\theta(\omega_0)$ is the initial condition of the phase at $t = 0$. After normalizing the time-varying response in (S9) with the phase-step amplitude, we took its derivative to calculate the impulse response in time domain. Finally, the phase domain transfer function of a resonator to changes in its driving frequency is calculated by taking the Laplace-transform of the impulse response as:

$$\Phi(s) = \mathcal{L}\left(\frac{1}{\tau}e^{-t/\tau}\right) = \frac{1}{\tau s + 1} \tag{S10}$$

Supplementary Note 2 Closed loop system function

We first model the resonator-PLL systems in phase domain using a commonly practiced 2nd-order Type-2 PLL [4] and the resonator response in (S10). We implement a model such that we can access the resonant frequency of the resonator as an input as well, by making the following modification:

$$\Phi(s) = \frac{1}{\tau s + 1} = 1 - \frac{\tau s}{\tau s + 1} \tag{S11}$$

which can be represented as shown in the blue box of Figure 1d. This model can then be further simplified by noting that the resonator has a positive feed-forward path that cancels with the PLL's negative feedback path. Furthermore, the PLL integrator cancels with the resonator differentiator. This allowed us to derive a simple, computationally tractable model (Supplementary Figure 2a) for the entire system. We derived the Laplace-domain transfer function of this closed loop system as:

$$H(s) = \frac{k_p s + k_i}{s^2 + s(1/\tau + k_p) + k_i} \tag{S12}$$

We first analyzed (S12) computationally by assuming different resonators for a given set of loop coefficients (solid lines in Figure 1e). We found that the resonator itself can substantially affect the system dynamics, which underlines the requirement of setting the loop coefficients for each resonator separately and carefully.

Supplementary Note 3 Tailoring the desired system response

In this work, for oscillating a resonator at multiple of its resonant modes simultaneously, we used a dedicated PLL in closed loop with each mode. In order to track the oscillation frequency of a mode precisely, we need a flat amplitude and linear phase response in the pass-band for minimum distortion of the frequency modulation signal and high enough rejection in the stop-band for minimum cross-talk between different modes and maximum noise rejection. Hence, we shall design the PLL such that we achieve a closed-loop transfer function identical to a Butterworth low-pass filter, since it has maximally flat gain and linear phase response. Since an nth order Butterworth filter includes n poles and no zeros in its transfer function, we start with the generalized system given in Supplementary Figure 2b utilizing an additional loop filter with $n-1$ poles in its forward path compared to the simplified model in Supplementary Figure 2a. We derived the closed loop transfer function of the system in Supplementary Figure 2b as:

$$H(s) = \frac{k_p s + k_i}{(s^2 + s/\tau) \prod_{k=1}^{n-1} (\tau_k s + 1) + k_p s + k_i} \tag{S13}$$

where τ_k are the poles introduced by the additional filter section in the forward path. Since there are one zero and $n+1$ poles in (S13), we can cancel the zero with one of the poles and position

the rest of the poles carefully to achieve an nth order Butterworth-type response (or similarly a Chebyshev-type or Elliptic response). We equate (S13) to a Butterworth filter transfer function with a 3-dB bandwidth, β and solve for the unknown parameters, k_p, k_i and τ_k.

$$H(s) = \frac{k_p s + k_i}{(s^2 + s/\tau)\prod_{k=1}^{n-1}(\tau_k s + 1) + k_p s + k_i} = \frac{1}{B_n(s/\beta)} \times \frac{k_p s + k_i}{k_p s + k_i} \tag{S14}$$

where B_n is the normalized Butterworth polynomial of nth order, which is given in [5]:

$$B_n(s) = \begin{cases} \prod_{k=1}^{\frac{n}{2}} \left[s^2 - s\ 2\cos\left(\frac{2k+n-1}{2n}\pi\right) + 1\right] & \text{if n is even} \\ (s+1)\prod_{k=1}^{\frac{n-1}{2}} \left[s^2 - s\ 2\cos\left(\frac{2k+n-1}{2n}\pi\right) + 1\right] & \text{if n is odd.} \end{cases} \tag{S15}$$

The solution of (S14) up to third order is given in Supplementary Table 1 along with the number of required poles in the additional section of the loop filter. Higher order parameters can also be calculated using symbolic toolbox of MATLAB, but the closed-form expressions are too long to be reported in this table. For loops higher than second order, the loop parameters are complex and can be implemented using digital filter design techniques. The closed loop transfer functions obtained by evaluating the calculated parameters in (S13) are given in Supplementary Figure 3.

In this work for simplicity and since the resonant mode frequencies are well separated, we implemented a first order system for driving the resonator modes using an array of PLLs. Without the need of the additional poles, the block diagram simplifies to that given in Supplementary Figure 2a. With the careful selection of the loop coefficients given in Supplementary Table 1, the system further simplifies to a first-order low pass filter with desired bandwidth.

Supplementary Note 4 Testing the system implementation

For testing the operation of the PLL system, we first developed a z-domain model of the PLL implementation on the FPGA board. The full-scale nonlinear system implementation is given in given in Supplementary Figure 2c. For z-domain analysis we approximated the multiplier in the PLL with a subtraction operation as signals are now in the phase domain, because the multiplier and low-pass filter effectively yield the phase difference between the input and the internal oscillator. We also omit the automatic gain control, as it operates only on the amplitude of the incoming signal, and does not affect the phase. We derived the z-domain response of the CIC filter [6] as:

$$H_{CIC}(z) = \left(\frac{1}{R}\sum_{k=1}^{R} z^k\right)^N \frac{1}{R}\frac{1-z^{-R}}{1-z^{-1}} \tag{S16}$$

where R and N are the rate factor and the order of the CIC, respectively. The second term is the transfer function of a zero-order hold [4] for modeling the effect of the rate change in the CIC filter.

Similarly, the loop filter and the NCO are represented by H_{LF} and H_{NCO}, respectively as follows:

$$H_{LF}(z) = \frac{T_S R k_i + k_p - k_p z^{-R}}{1 - z^{-R}} \tag{S17}$$

$$H_{NCO}(z) = \frac{T_S}{1 - z^{-1}} \tag{S18}$$

where T_S is the sampling period of the fastest rate in the loop, which is 10 ns in this case. Note that the CIC filter and the loop filter operates at a slower rate than the NCO. Furthermore, we modeled the resonator response (S11) in z-domain as follows:

$$H_{resonator}(z) = \frac{\tau\alpha}{T_s} \frac{1 - z^{-1}}{1 + (\alpha - 1)z^{-1}} \tag{S19}$$

where α is $T_s/(\tau + T_s)$.

To measure the response of our PLL, we add a 10 degree phase step (a frequency pulse of about 2.7MHz for 10 ns) to the NCO and then observe the response of the NCO frequency. To ensure that our PLL implementation and its z-domain model works properly, we tested the PLL over a 9x9x3 grid of the following parameters: the proportional path gain k_p, the integral path gain k_i, and the CIC filter bandwidth, parameterized as the rate change factor, R. We then plot the Fourier transforms of the observed impulse responses against the predicted frequency responses from the z-domain PLL model with those particular settings, as shown for several parameter sets in Supplementary Figure 4. Then we analyzed the system with various loop coefficients with the resonator in closed loop. We found that the operation of the resonator-PLL system and the model predictions show excellent agreement.

Supplementary Note 5 Frequency content of the particle signals

To determine the necessary bandwidth for each resonator mode, we calculate the frequency modulation signal given in [1] for a particle moving at uniform speed towards the tip of the cantilever and then returning back to the base. We then calculate the cumulative energy density for varying frequencies and determine the frequency at which 99.99% of signal energy will be retained (Supplementary Figure 5). For a 100 ms peak, these frequencies are roughly 70, 150, 185 and 210 Hz, respectively. Conservatively, we set the 3 dB bandwidth of our resonator-PLL system to a little over twice these values, yielding bandwidths of 150, 335, 435 and 505 Hz for the transfer functions shown in Figure 2b.

Supplementary Note 6 Precision of the position estimation

We calculated the root-mean-square (RMS) error of the position estimation of a 30 fg ($\sim$ 150 nm gold) particle as a function of the position along the resonator length by using the fitting algorithm

described in the Methods section. We added experimentally measured noise waveforms to the simulated signals of identical particles passing through the resonator in 150 ms. Then, we estimated the position of each particle throughout their 150-ms transits. We calculated the RMS error of positions estimated by the fitting algorithm when the signals from first two, three and four modes as a function of particle position (Supplementary Figure 6a). The results show that the number of modes improves the precision of the position estimation.

It is known that the mass sensitivity of a mechanical resonator improves with miniaturization. In order to determine the effect of miniaturization on position precision for less dense, biologically relevant nanoparticles, we calculated the precision that could be achieved by a hypothetical SNR device utilizing a 20 μm long, 0.5 μm thick and 5 μm wide cantilever with an integrated fluidic channel (250 nm by 1 μm in cross-section). We calculate the fundamental resonant frequency of such a cantilever when it is filled with water as 1.8 MHz. Currently, our oscillator system can operate with 5 ppb frequency stability around 1.8 MHz [7]. We assumed with improved transduction techniques and high-frequency (HF) and very-high-frequency (VHF) control electronics, the same stability level could be achieved for the higher order modes as well. Under these assumptions we calculated the position precision for a human immunodeficiency virus, HIV (80-attogram buoyant mass and $\sim$100 nm size) using the same algorithm for estimating the particle position that was used for Fig. 5 of the main text. To demonstrate the effect of the increased number of modes, we expoited the first two, four and eight modes of the hypothetical SNR for the calculations. The highest frequency of operation for these three cases are 11, 61, 281 MHz, respectively. The resulting position precision is plotted in Supplementary Figure 6b.

Supplementary References

[1] S. Dohn, W. Svendsen, A. Boisen, and O. Hansen, "Mass and position determination of attached particles on cantilever based mass sensors," *Review of Scientific Instruments*, vol. 78, p. 103303, Oct. 2007.

[2] T. R. Albrecht, P. Grtter, D. Horne, and D. Rugar, "Frequency modulation detection using high-Q cantilevers for enhanced force microscope sensitivity," *Journal of Applied Physics*, vol. 69, no. 2, p. 668, 1991.

[3] K. Kobayashi, H. Yamada, and K. Matsushige, "Frequency noise in frequency modulation atomic force microscopy," *Review of Scientific Instruments*, vol. 80, p. 043708, Apr. 2009.

[4] F. M. Gardner, *Phaselock Techniques*. Hoboken, NJ: John Wiley and Sons, 3 ed., 2005.

[5] S. Butterworth, "On the Theory of Filter Amplifiers," *The Wireless Engineer*, pp. 536–541, 1930.

[6] E. Hogenauer, "An economical class of digital filters for decimation and interpolation," *IEEE Transactions on Acoustics, Speech and Signal Processing*, vol. 29, pp. 155–162, Apr. 1981.

[7] S. Olcum, N. Cermak, S. C. Wasserman, K. S. Christine, H. Atsumi, K. R. Payer, W. Shen, J. Lee, A. M. Belcher, S. N. Bhatia, and S. R. Manalis, "Weighing nanoparticles in solution at the attogram scale," *Proceedings of the National Academy of Sciences*, vol. 111, pp. 1310–1315, Jan. 2014.
